# A Reverse Time-Course Method for Transcriptional Chase Analyses of mRNA Half-Lives in Cultured Cells

**DOI:** 10.1371/journal.pone.0040827

**Published:** 2012-07-13

**Authors:** Osheiza Abdulmalik, Alyssa A. Lombardi, J. Eric Russell

**Affiliations:** 1 Department of Pediatrics (Hematology), University of Pennsylvania School of Medicine and The Children’s Hospital of Philadelphia, Philadelphia, Pennsylvania, United States of America; 2 Department of Medicine (Hematology-Oncology), University of Pennsylvania School of Medicine and The Children’s Hospital of Philadelphia, Philadelphia, Pennsylvania, United States of America; UMDNJ-New Jersey Medical School, United States of America

## Abstract

Standard methods for assessing mRNA stabilities in intact cells are labor-intensive and can generate half-life (t_1/2_) measures that are both imprecise and inaccurate. We describe modifications to a conventional tetracycline-conditional transcriptional chase method for analyzing mRNA stability that significantly simplify its conduct, while generating highly reproducible and accurate t_1/2_ values. The revised method–which is conducted as a reverse time course, and which accounts for interval expansion in the number of cultured cells–is validated for the analyses of mRNAs with both short and long half-lives. This approach facilitates accurate assessment of mRNA metabolism, providing a user-friendly tool for detailed investigations into their structures and functions, as well as the processes that contribute to their post-transcriptional regulation.

## Introduction

The biological importance of regulated mRNA stability is illustrated by pathological consequences that follow the abnormal metabolism of individual transcripts. A number of medical conditions have been linked to changes in the stabilities of specific mRNAs, including Type 2 Gaucher disease (glucocerebrosidase) [1], ichthyosis vulgaris (profilaggrin) [2], age-related macular degeneration (ARMS2) [3], systemic lupus erythematosis (TCR-ζ) [4], and breast cancer (cyclin D1) [5]. The broader implications of post-transcriptional regulatory processes are illustrated by expression-profiling analyses of T-lymphocytic cells suggesting that nearly one-half of inductive changes in gene expression result from alterations in mRNA stability [6].

The general utilities of *in vitro* and cultured cell approaches that are commonly employed to estimate mRNA-specific t_1/2_ values are limited by important methodological considerations. Assays conducted in cell-free systems–though convenient–fail to account for translation-coupled effects on mRNA metabolism [7] and are unlikely to faithfully reproduce stable and/or transient mRNA-protein interactions that specify the kinetics of mRNA decay in intact cells. Different concerns apply to cultured cell analyses that quantitate the temporal decline in a study mRNA following transcriptional silencing of its cognate gene. Commonly used transcriptional inhibitors–actinomycin D and DRB (5,6-dichloro-beta-D-ribofuranosyl benzimidazole)–are global and nonspecific in effect, altering expression of the study mRNA, as well as the expression of mRNA-stabilizing and -decay factors that may be required for its constitutive regulation. Other methods that permit gene-specific transcriptional regulation in intact cells (e.g., serum stimulation of *fos* promoter-linked genes [8]) induce alterations in the cellular milieu that may have equally unpredictable effects on the observed half-lives of study mRNAs.

**Figure 1:**
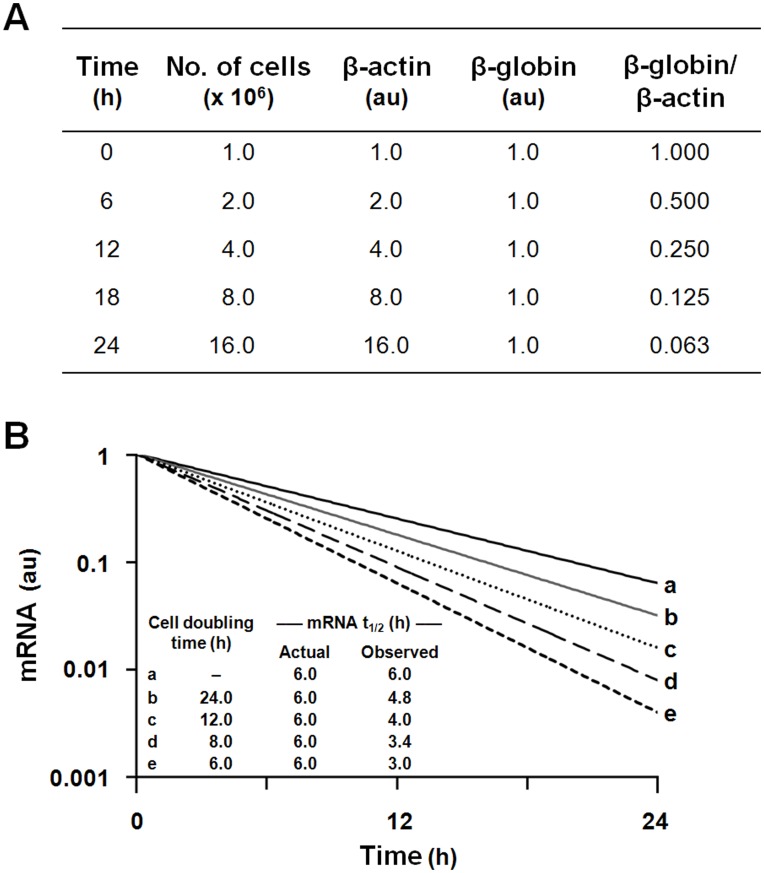
Effects of cell doubling time on the apparent t_1/2_ value of a study mRNA. (A) Hypothetical mRNA decay. Cell aliquots, each originally containing 1.0×10^6^ cells, are incubated for defined intervals between 0 and 24 hours. Cells in each aliquot double every 6 hours, in parallel with the total amount of β-actin mRNA. At T = 0, each aliquot contains an equal quantity [1 arbitrary unit (au)] of an infinitely stable β-globin mRNA, encoded by a gene that has been transcriptionally silenced. The decline in the ratio of the globin:actin mRNAs, which erroneously indicates a half-life value of 6 hours for the globin mRNA, fails to account for interval expansion in cell number. These same principles apply to mRNAs with finite stabilities (panel B). (B) True and uncorrected t_1/2_ values in cells with different doubling times. Curves illustrate the uncorrected half-life for a test mRNA, following transcriptional silencing of its encoding gene, if the interval expansion in cell number is not considered. The examples utilize an mRNA with a true t_1/2_ = 6 h, expressed in cells that are growth arrested (curve *a*) or display doubling times of 24, 8, 6, or 4 hours (curves *b* through *e*, respectively).

Tetracycline-conditional (‘on-off’) transcriptional chase approaches for assessing mRNA half-lives are widely employed because they are conducted in intact cells, are mRNA-specific, and do not alter cellular homeostasis. These analyses require cells that express a tetracycline trans-activator (tTA) fusion protein that promotes transcription of a second, exogenous study gene linked to a compound tetracycline response element (TRE) [9]. The transcription of TRE-linked genes is rapidly inhibited by exposure to antibiotics [tetracycline (tet) or doxycycline (dox)] that bind and inactivate tTA. Half-life analyses are conducted in tTA-transfected cells that express a TRE-linked study gene, by amending cultures with tet or dox and sacrificing aliquots at defined intervals. The level of study mRNA in each aliquot is then quantitated, relative to the level of an antibiotic-indifferent control mRNA, using any of several methods. When properly conducted, this transcriptional chase strategy additionally requires the accurate enumeration of cells in each individual aliquot, to permit correction for the expansion in cell number–and corresponding increase in the level of tet-indifferent control mRNA–that occurs during the interval when the study gene is transcriptionally silent. This concept is illustrated by considering that, in the absence of this correction, an infinitely stable study mRNA encoded by a transcriptionally-silenced gene would appear to have a t_1/2_ value equal to the doubling time of the cell culture (Fig. 1). As a practical matter, the efforts required to mobilize and subsequently count cells in individual aliquots significantly limit the number of experimental replicates that can be conducted and/or the frequency with which data points can be collected. These low-throughput imperatives mandate data sets that are frequently too small to produce statistically robust estimates of mRNA half-life.

We reasoned that technical barriers to the broader application of tet-conditional RNA half-life analyses could be resolved by altering both the design of the transcriptional chase, as well as the manner used to correct normalized study mRNA values for interval expansion in cell number. In the conventional method, identical aliquots are simultaneously amended with antibiotic, and individually processed (trypsinized, resuspended, counted, and transferred to storage) at defined intervals. Normalized RT-qPCR values for the study mRNA are then corrected for the corresponding aliquot-specific cell number, the data regressed to an exponential function, and a half-life value calculated. The new reverse-chase (RC) method uses a reverse time-course approach in which identical aliquots are individually amended with antibiotic at defined intervals, and simultaneously transferred to storage at the conclusion of the experiment. Normalized values for the study mRNA are first regressed to an exponential function, which is subsequently corrected to a formal t_1/2_ value using an experimentally determined culture-specific factor describing the rate of cell growth.

## Results

The reverse-chase strategy for assessing mRNA stability was designed to mitigate the throughput-limiting characteristics of the conventional method that is commonly used for this purpose. The RC approach eliminates the effort required for conventional, iterative processing of individual aliquots, thus facilitating the generation of data sets with replicate and/or closely spaced time points. An additional benefit of the RC strategy is that it provides for equal numbers of cells in all aliquots at the time of sacrifice (unlike the conventional approach), obviating the need to individually adjust samples to maintain control mRNA values within the linear range of RT-qPCR or other quantitation assays.

**Figure 2:**
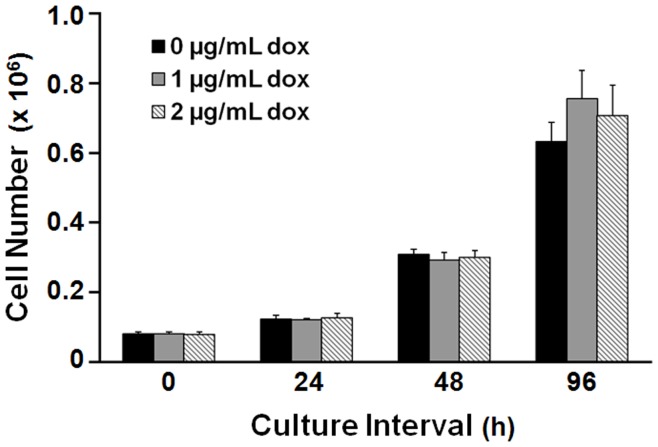
HeLa cell growth is not affected by doxycycline supplement. Values are the mean of three independent cultures; error bars indicate ±1 S.E.

We tested the RC method using tTA-expressing HeLa cells engineered to stably express TRE-linked genes encoding human β-globin mRNAs (not shown). The expansion of cultured HeLa cells was unaffected by supplemental dox, confirming the expectation that aliquots in RC analyses would each contain the same number of cells, regardless of antibiotic-exposure interval (Fig. 2).

**Figure 3:**
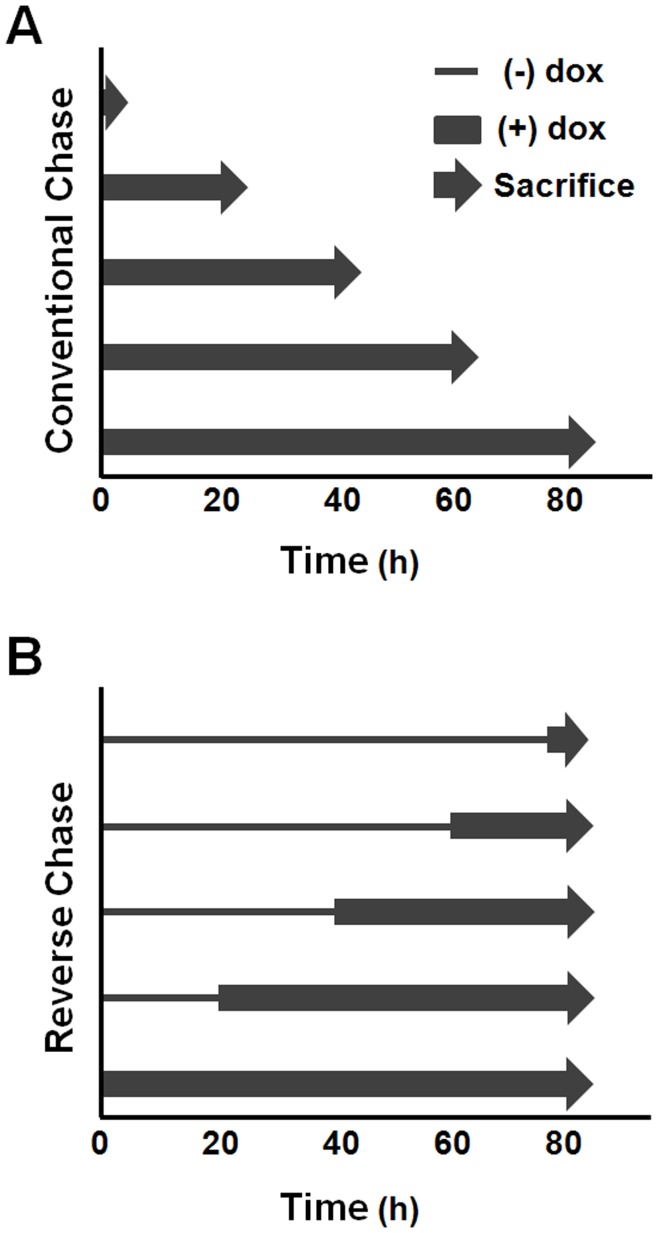
Experimental schemata for transcriptional chase analyses of mRNA stability. (A) Conventional chase method. Identical aliquots cultured in doxycycline (dox)-supplemented media (thick line) are sacrificed at defined intervals (arrowheads). A hypothetical 80-hr chase experiment is illustrated. (B) Reverse-chase method. Identical aliquots cultured in dox-free media (thin line) are amended with dox at defined intervals, and sacrificed simultaneously at the conclusion of the experiment.

**Figure 4:**
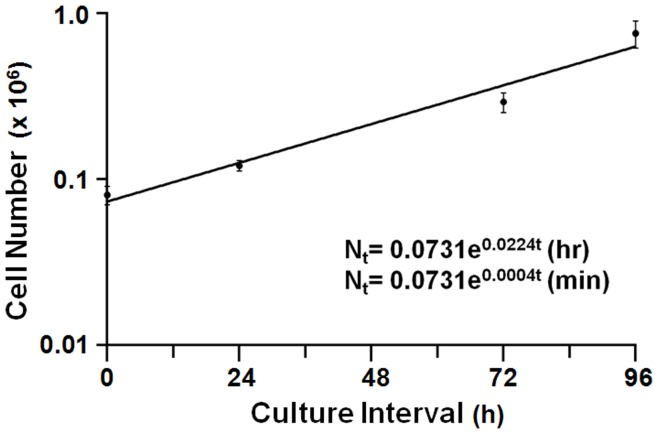
HeLa cell expansion under transcriptional chase conditions. Data from dox-supplementation experiments in Fig. 2 (1 µg/mL) was regressed to an exponential function, and expansion factors defined [j = 0.0224 (h) or 0.0004 (min)].

**Figure 5:**
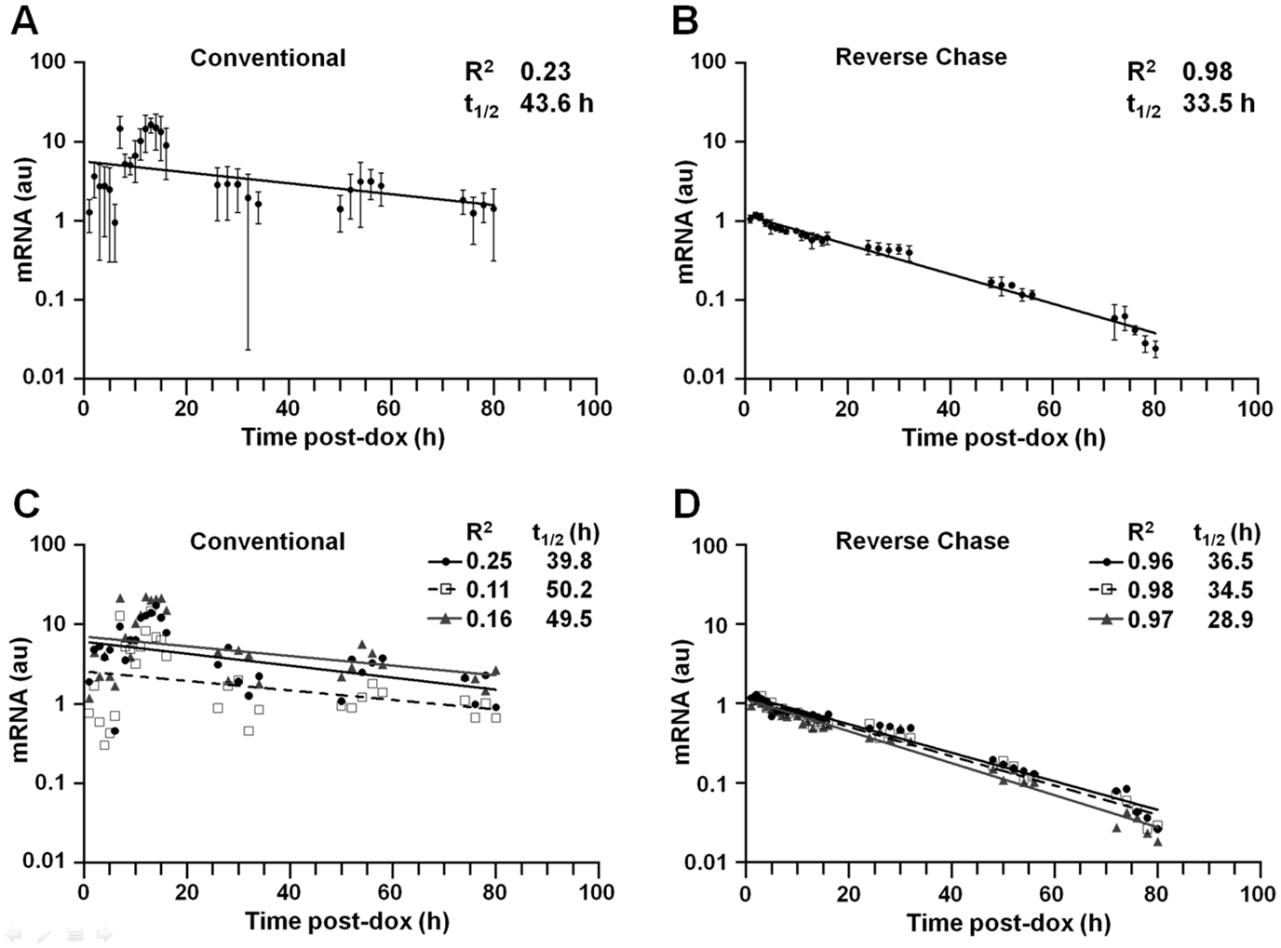
Comparative transcriptional chase analyses of a long-lived test mRNA. (A) Aggregate analysis using the conventional method. Cell aliquots were amended with dox at t_0_, and sacrificed at defined intervals. Levels of β^WT^ mRNA were determined by RT-qPCR relative to control dox-indifferent β-actin mRNA, using the ΔΔCt method. Normalized RT-qPCR values for β^WT^ mRNA were corrected for aliquot-specific cell numbers, then plotted. Points represent the mean ± S.D. from three replicate experiments. A t_1/2_ value was calculated from the exponential decay constant corresponding to the best-fit curve. (B) Aggregate analysis using the reverse-chase method. Cell aliquots were amended with dox at defined intervals and sacrificed simultaneously at t = 80 h. Normalized values for β^WT^ mRNA were determined by RT-qPCR, then plotted. Points represent the mean ± S.D. from three replicate experiments. A t_1/2_ value was calculated from the exponential decay constant corresponding to the best-fit curve, corrected for an expansion factor describing the growth rate of cultured HeLa cells. (C) Analyses of individual replicates using the conventional method. Normalized values for each of three biological replicates reported in panel A were corrected for the number of cells present in each aliquot at the time of sacrifice, and t_1/2_ values calculated. (D) Analyses of individual replicates using the reverse-chase method. Normalized values for each of three biological replicates reported in panel B were directly plotted, and t_1/2_ values calculated following correction for interval cell expansion.

Conventional and RC stability analyses were subsequently conducted in parallel in cells expressing wild-type β-globin mRNA (β^WT^). For the conventional method, identical cell aliquots were simultaneously supplemented with dox (t = 0) and triplicate samples sacrificed, with cell counting, at defined intervals for 80 hours (Fig. 3A). For the RC method, identical cell aliquots were amended with dox at defined intervals, and simultaneously sacrificed, without cell counting, at 80 hours (Fig. 3B). In both experiments, β^WT^ mRNA was quantitated by RT-qPCR relative to dox-indifferent control β-actin mRNA. Half-life values for the β^WT^ mRNA were subsequently calculated from normalized RT-qPCR results using either aliquot-specific cell numbers (conventional method), or an experimentally determined factor (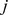, see Methods) that describes the rate at which each cell culture is expands (Fig. 4). Although both the conventional and RC studies generated equal numbers of data points, the conventional method specified a decay curve with a high level of uncertainty (R^2^ = 0.23; Fig. 5A), while the RC results regressed to a highly reliable exponential decay function (R^2^ = 0.98; Fig. 5B). Plotted separately, the three conventional replicates displayed low R^2^ values (0.11–0.25; Fig. 5C), while the three RC replicates exhibited correspondingly high R^2^ values (0.96–0.98; Fig. 5D). As might be anticipated, the conventional method produced t_1/2_ values for the study mRNA that were poorly reproducible (range 39.8–50.2 h), in contrast to t_1/2_ values calculated from the RC method (range 28.9–36.5 h).

**Figure 6:**
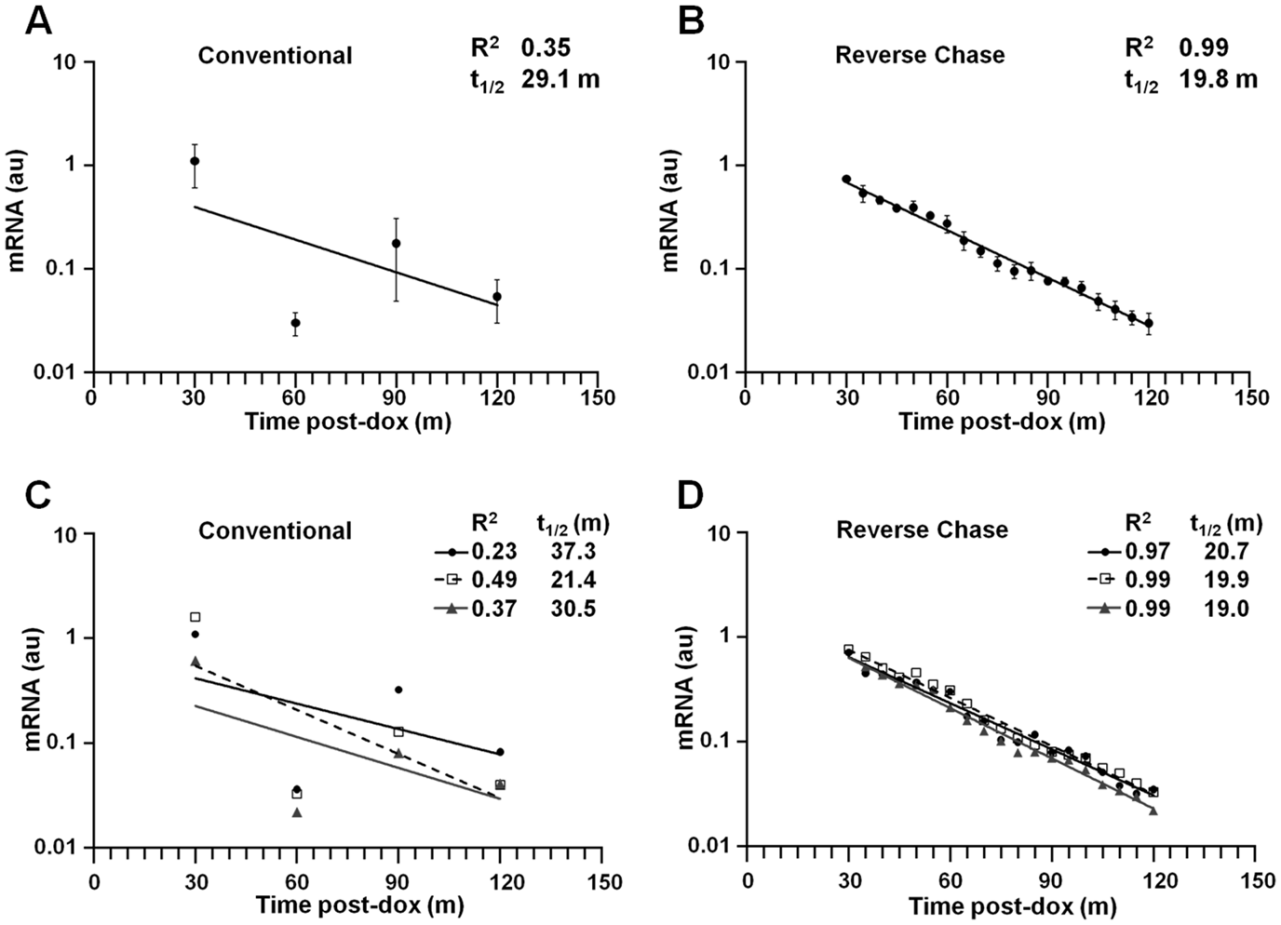
Comparative transcriptional chase analyses of a short-lived study mRNA. tTA-expressing HeLa cells were engineered to stably express a short-lived derivative human β-globin mRNA (β^ARE^). Experiments were conducted as described in Fig. 3, including the calculation of mRNA t_1/2_ values. (A) Aggregate analysis using the conventional method. Cell aliquots were amended with tet at t_0_, and sacrificed at 30-min intervals. Points represent the mean ± S.D. from three replicate experiments. (B) Aggregate analysis using the reverse-chase method. Cell aliquots were amended with tet at 5-min intervals and sacrificed simultaneously after 120 min. Points represent the mean ± S.D. of three independent experiments. (C, D) Analyses of individual replicates. Curves illustrate results from individual replicates that were analyzed in aggregate in panels A and B. Data are presented as described in Fig. 3. (C) Conventional method. (D) Reverse-chase method.

Similar comparative analyses demonstrated the superiority of the RC method for analyses of mRNAs with shorter half-life values. The stability of a derivative β-globin mRNA (β^ARE^) containing a destabilizing AU-rich element within its 3′UTR [12] was studied over a 120-minute chase interval in tTA-expressing HeLa cells using both the conventional and RC approaches. Cell aliquots were sacrificed with cell counting at 30-minute intervals (for the conventional method) or without counting at five-minute intervals (RC method). The conventional approach yielded four averaged time points, while the RC protocol permitted the accumulation of 21 averaged time points. The RC data regressed to a more reliable exponential decay function when replicates were analyzed in aggregate [R^2^ = 0.99 (RC) *v* 0.35 (conventional); Fig. 6A and B) or individually [R^2^>0.97 (RC) *v* 0.23–0.49 (conventional); Fig. 6C and D]. Likewise, mRNA t_1/2_ values calculated from the RC data were highly reproducible (range 19.0–20.7 min), in contrast to values determined by the conventional method (range 21.4–37.3 min). Conventional analyses displayed a high variability–even when uncorrected for cell number–emphasizing that simultaneous sacrifice can impart high reproducibility to data collected using the RC method (not shown).

## Discussion

The reverse-chase strategy resolves critical limitations of conventional tetracycline chase experiments: substantial hands-on time that restricts data acquisition, and negatively impacts both data reliability and reproducibility. For analyses requiring a prolonged chase interval–where changes in cell number can be significant–conventional cell sacrifice and counting requires as much as 30 minutes of attention per time point, even for a limited number of samples. In contrast, the RC method requires 10–15 seconds of attention per time point (for the addition of antibiotic to individual aliquots) substantially increasing the number of samples that can be evaluated. Moreover, because the RC strategy permits simultaneous sacrifice of all aliquots at the conclusion of the chase interval, concentrations of mRNA (which correspond with cell number) are equal, and do not need to be individually adjusted to stay within the linear range of the mRNA quantitation method. Although not illustrated here, the denser data set obtained by the RC method also permits the application of statistical tools that account for the effect of background dox-indifferent transcription on the calculated mRNA half-life [13], [14]. We have also observed that the RC method displays an almost negligible inter-operator variability, which is critically important for the conduct of prolonged chase experiments requiring investigators to work in two or more shifts (not shown).

One seemingly minor limitation to the RC method is that it requires study mRNAs to be expressed in steady state; i.e., transcribed from TRE-regulated genes that are stably integrated in tTA-expressing cells. We have not found this to be a major impediment, as a large number of tTA-expressing cell lines are commercially available [15] or have been previously generated by individual investigators [12], [16]. Moreover, since all three components required for tet-regulated transcription are exogenous (tTA transactivator, TRE-linked study gene, and tetracycline), cell-homologous systems for studying the properties of a specific mRNA can easily be engineered from nearly any existing cultured cell line. Consequently, the method that we describe is not restricted to HeLa cells, but can be applied to cell lines that model many normal or diseased tissues.

We have also observed that half-life analyses conducted in cells that transiently express TRE-linked study genes are less reliable, and less reproducible, than similar analyses conducted in a corresponding cell line that stably expresses the study gene. Among other limitations, transient expression analyses utilize cells recovering from methodological insults (electroporation, lipofection) that alter cell homeostasis; do not account for cell-to-cell variation in the expression of study mRNA; and cannot guarantee a consistent baseline level of the study mRNA in independent experiments. In contrast, cell lines that *stably* express the test mRNA are not subject to any of these limitations: the test mRNA is expressed at a defined level, at steady-state, in healthy, clonal cells. As a matter of practice, we typically use transient assays for pilot, low-resolution analyses, and subsequently generate stable cell lines for definitive, reproducible and highly reliable measures of mRNA stability. This approach determines mRNA half-live values that are reliable and highly reproducible, and is likely to lower the ‘effort barrier’ for RNA biologists and other investigators who, until now, have avoided definitive mRNA stability analyses in stable cells because of the corresponding technical challenges.

## Methods

### Plasmids

The construction of parental pTRE-β^WT^, which contains the full-length human (h) β-globin gene on a 3.3-kb fragment of genomic DNA, has been previously described [10]. pTRE-β^ARE^ was generated from pTRE-β^WT^ by insertion of a 59-bp AU-rich mRNA instability element [11] at a position 15 bp 3′ to the translation stop codon [10]. A hygromycin-resistance gene, encompassed by a 1.5-kb *Xho*I fragment of pTRE2hyg (Clontech), was subsequently ligated into pTRE-β^WT^ and -β^ARE^ at the corresponding position.

### Cell Culture

HeLa cells expressing the tetracycline *trans*-activator (tTA) fusion protein (HeLa Tet-Off cell line, Clontech) [15] were maintained in DMEM/F12 media supplemented with 10% fetal bovine serum and antibiotics. Cells in log-phase growth were transfected with pTRE-β^WT^ or -β^ARE^ using Superfect reagent according to the manufacturer’s recommendations (Qiagen). Hygromycin-resistant clones were isolated using standard cloning-disc methodology, and subsequently screened for steady-state levels of β-globin mRNA in tetracycline-free media.

### Tetracycline-conditional Gene Silencing

#### Conventional method

HeLa cells (1×10^4^) were aliquoted in 450 µL media in 24-well format (for 120-minute chase experiments), or 225 µL in a 48-well format (for 80-hr chase experiments) 24 hours prior to the start of the transcriptional chase. At t = 0, all aliquots were supplemented with a 10X stock solution of doxycycline (10 µg/µL in culture media).

#### Reverse Chase method

HeLa cells (5×10^3^) were aliquoted in 180 µL media in a 96-well format 24 hours prior to the start of the transcriptional chase. Starting at t = 0, and continuing for the duration of the experiment, serial aliquots were amended with a 10X doxycycline stock at defined intervals. For both the 80-hr conventional and reverse-chase methods, doxycycline was refreshed at 48 hours with a second volume of 10X stock.

### Cell Sacrifice

#### Conventional method

Cells were washed twice with excess phosphate-buffered saline (PBS), mobilized with trypsin, washed, and re-suspended in 30 µL of PBS. Ten µL of the suspension was reserved for cell counting, which was conducted in triplicate using a hemocytometer, and reported as a mean value. Whole-cell lysates were prepared from the remaining 20 µL using the Cells-to-Ct kit (Applied Biosystems).

#### Reverse-chase method

Wells were washed twice with excess PBS and lysed *in situ* using Cells-to-Ct reagents.

#### Analysis of cell growth rate

Approximately 5×10^4^ HeLa cells were cultured in 12-well plates in the presence or absence of dox, then manually counted at defined intervals. 96-hour aliquots were refreshed with dox-supplemented media at t = 48 hr.

#### RT-qPCR

Equal volumes of prepared RNA were subjected to RT using Cells-to-Ct reagent. First-strand cDNA was subjected to qPCR amplification using Taqman assays specific for β-globin (#00747223_g1) or endogenous control β-actin (#99999903_m1) mRNAs, according to the manufacturer’s recommendations (Applied Biosystems). Samples were analyzed using an ABI 7500 Real-Time PCR system (Applied Biosystems). Each RT-qPCR assay was conducted in triplicate using a previously validated multiplexed format (not shown); samples exhibiting outlier Ct values (defined by Applied Biosystems) were censored.

### Data Analysis

#### Conventional method

For each sample, the level of globin mRNA was first normalized to the level of control β-actin mRNA, and subsequently to the aliquot-specific cell number. These data were regressed to an experimental decay function, and half-life values determined.

#### Reverse-chase method

Levels of globin mRNA were normalized to the level of control β-actin mRNA, then regressed to an exponential decay function. Decay factors were adjusted for increases in cell number using an experimentally determined expansion factor (see below).

### Correction of RT-qPCR Data for Expansion of Cell Number

Results from RT-qPCR analyses are fit by nonlinear regression to the general equation for exponential decay:
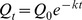
(1)where 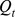 and 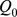 are observed quantities of subject mRNA normalized to levels of β-actin mRNA at time = 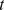 and time = 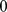, and 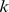 is the decay constant. A new term 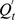 accounts for the increase in cell number during the interval when TRE-regulated genes are transcriptionally silent. 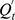 is derived from 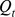 by multiplying by 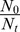, where 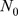 and 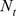 are the number of cells present at time = 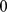 and time = 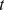, respectively:



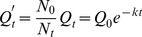
(2)The equation can be solved by manual determination of 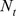 at each individual time = 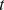 (as required by the conventional method), or by separately determining the expansion constant 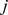 that describes the growth of specific cells under defined culture conditions (as practiced in the RC method). Specifically,
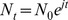
(3)


Substituting for 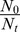:



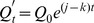
(4)A t_1/2_ value for the test mRNA is calculated as 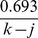 using experimentally determined values for both 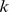 and 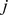. Values for 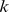 and 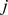 derived from our experiments are included in Table 1.

**Table 1:** Values used in the current report.

Replicate	*_j_*	*_k_*	*_k−j_*	t_1/2_
ARE 1	.0004	.0338	.0334	20.7 m
ARE 2	.0004	.0353	.0349	19.9 m
ARE 3	.0004	.0369	.0365	19.0 m
WT 1	.0224	.0414	.0190	36.5 h
WT 2	.0224	.0425	.0201	34.5 h
WT 3	.0224	.0464	.0240	28.9 h
